# Morphological and Molecular Characterization of *Quinisulcius curvus* from China

**DOI:** 10.21307/jofnem-2021-086

**Published:** 2021-11-03

**Authors:** Jianfeng Gu, Maria Munawar, Pablo Castillo, Bo Cai

**Affiliations:** 1 Ningbo Customs Technology Centre (Ningbo Inspection and Quarantine Science Technology Academy), Ningbo, Zhejiang 315100, P. R. China; 2 Department of Biological Sciences, University of Lethbridge, 4401 University Drive West, Lethbridge, AB, T1K 3M4, Canada; 3 Institute for Sustainable Agriculture (IAS), Spanish National Research Council (CSIC), Campus de Excelencia Internacional Agroalimentario, ceiA3, Avenida Menéndez Pidal s/n, 14004 Córdoba, Spain; 4 Hainan Province Engineering Research Center for Quarantine, Prevention and Control of Exotic Pests, Haikou Customs District, Haikou, Hainan 570311, P. R. China

**Keywords:** DNA sequencing, Morphology, Morphometrics, New record, Phylogeny, Stunt nematode, Subfamily Telotylenchinae

## Abstract

A stunt nematode species, *Quinisulcius curvus*, recovered from the rhizosphere of sea randa (*Guettarda speciosa*), is described and characterized herein based on integrative taxonomy. Morphometrics and distribution of all reported populations of *Q. curvus* are also discussed. The Chinese population of *Q. curvus* displayed slight variation in stylet length; however, the rest of the characters matches well with the original description. This is the first record of *Q. curvus* from Hainan, China, and the first molecular characterization for this species. Phylogenetic analysis based on partial 18S, 28S and ITS sequences placed *Q. curvus* with related stunt nematodes species, but clearly separated from them. The present study expanded the geographic record and provided molecular data on *Q. curvus* from China.

The members of subfamily Telotylenchinae comprised of large group of plant-parasitic nematodes which commonly known as stunt nematodes (Handoo et al., 2014). Based on the recent taxonomic scheme of Geraert (2011), the subfamily Telotylenchinae includes nine genera: *Histotylenchus* (Siddiqi, 1971); *Neodolichorhynchus* (Jairajpuri and Hunt, 1984); *Paratrophurus* (Arias, 1970); *Quinisulcius* (Siddiqi, 1971); *Sauertylenchus* (Sher, 1974); *Telotylenchus* (Siddiqi, 1960); *Trichotylenchus* (Whitehead, 1960); *Trophurus* (Loof, 1956); and *Tylenchorhynchus* (Cobb, 1913). Among these, the genus *Quinisulcius* was initially proposed by Siddiqi (1971) for those *Tylenchorhynchus* who have five lines in the lateral field and modified the generic definition by adding more diagnostic characters. Tarjan (1973) accepted the genus and enhanced the species resolution by providing the key to valid species of *Quinisulcius*.

Since then, there have been several opinions on taxonomic status of *Quinisulcius,* it was either synonymized to *Tylenchorhynchus* (Brzeski and Dolinski, 1998; Esser, 1991; Fortuner and Luc, 1987) or recognized as valid and independent genus in subfamily Telotylenchinae (Geraert, 2011; Handoo, 2000; Siddiqi, 2000). Currently the genus contains 17 valid species that have been reported from the USA or from the Asian countries. Once formally described, only *Q. acutus* (Allen, 1955) Siddiqi, 1971; *Q. capitatus* (Allen, 1955) Siddiqi, 1971 and *Q. curvus* (Williams, 1960) Siddiqi, 1971 were reported outside of their type locality (Geraert, 2011; Munawar et al., 2021).

Unlike *Tylenchorhynchus* species, the biology and host association of *Quinisulcius* species are not well documented. So far, *Q. capitatus* and *Q. acutus* have been found damaging agronomic and horticultural crops including melon, maize, wheat, and sorghum (Claflin, 1984; Cuarezma-Teran and Trevathan, 1985; Khan et al., 1988; Khan and Khanzada, 1990; Singh et al., 2013; Todd et al., 2014). Therefore, it is imperative to correctly diagnose, and document the species identity in order to recognize the host association and geographic range of the species in question. In addition to that, information regarding the occurrence and distribution of plant-parasitic nematodes in agricultural or forestry areas is important to assess the damage potential of inhabiting species (Hafez et al., 2010). In majority of cases, nematode damage symptoms are frequently underestimated or misidentified to other stresses (Barker et al., 1994; Singh et al., 2013).

The nematode diversity is not well studied in Hainan Province, since the majority of nematological research was conducted on the more aggressive plant parasitic nematodes such as root-knot nematodes (Liu et al., 2005; Long et al., 2019; Zhuo et al., 2008), cyst forming nematodes (Zhuo et al., 2013) and virus vector nematodes (Li et al., 2020; Luo et al., 2001).

Therefore, detailed samplings were conducted with a focus to determine the identity of ectoparasitic nematodes in Hainan Province. In this study, a population of stunt nematodes was recovered from the rhizosphere of a shrub, sea randa in 2019. No above ground symptoms were observed on the host. The population was detected in high density (200-300/100 g of soil) as compared to other soil nematodes. Preliminary examination showed that the species has five lateral lines which is a salient characteristic of genus *Quinisulcius*. Therefore morphological and molecular characterization were performed, and the results were compared with the nominal species of *Quinisulcius*. The morphological characters of the population confirm the close resemblance to *Quinisulcius curvus* (Williams, 1960) Siddiqi, 1971. Literature studies indicated that the species was described decades ago in the rhizosphere of Sugarcane from Mauritius with scarce morphological details. It also elaborated that *Q. curvus* was reported from Dominican Republic, Pacific Islands, Martinique, Thailand, India but without morphometrical or morphological characterization (Bohra and Baqri, 2005; Bridge, 1988; Cadet et al., 1994; Román, 1968; Toida et al., 1996), moreover some reports are in languages other than English. Besides, we also noted that of 17 nominal species, sequence-based information of *Quinisulcius* is only available for *Q. capitatus*. Therefore, the objective of the present study was i) to provide detailed morphological, morphometrical and molecular characterization of *Q. curvus* ii) study the phylogenetic relationships of *Q. curvus* with other stunt nematode species.

## Materials and methods

### Isolation and morphological observation of nematodes

Rhizosphere soil samples were collected from sea randa. Nematodes were extracted from soil and root samples of sea randa using the modified Cobb sieving and flotation-centrifugation method (Jenkins, 1964). Nematode suspension contained mixture of herbivores (Dorylaimids), fungivores (*Aphelenchoides*, *Filenchus*, *Aphelenchus* spp.) bacterivores (*Rhabditis* sp.) and populations of spiral and *Quinisulcius* sp. Since *Quinisulcius* was the most abundant species in the soil suspension, the females were collected individually from the mixture of soil nematodes and studied under light micrscope. For preliminary examinations, fresh *Quinisulcius* females were transferred to a drop of distilled water, heat relaxed and observed under a Zeiss microscope. For additional morphological and morphometric studies, nematodes were killed and fixed in hot formalin (4% formaldehyde) and processed to ethanol-glycerin dehydration according to Seinhorst (1959) as modified by De Grisse (1969) and mounted on permanent slides. Measurements were made on mounted specimens, light micrographs and illustrations were produced using a Zeiss microscope equipped with a Zeiss AxioCam MRm CCD camera.

### DNA extraction, PCR and sequencing

DNA samples were prepared according to Li et al. (2008). Four sets of primers (synthesis by Invitrogen, Shanghai, China) were used in the PCR analyses to amplify sequences of the near full-length 18S region, D2-D3 expansion segments of 28S, and ITS of ribosomal RNA genes (rDNA). The 18S region was amplified with primers 988F/1912R and 1813F/2646R (Holterman et al., 2006). The 28S D2-D3 region was amplified with primers D2A/D3B (De Ley et al., 1999), and the ITS was amplified using primers TW81/AB28 (Tanha Maafi et al., 2003). PCR conditions were as described by Ye et al. (2007) and Li et al. (2008). PCR products were separated on 1% agarose gels and visualized by staining with ethidium bromide. PCR products with high quality were purified for cloning and sequencing by Invitrogen, Shanghai, China.

### Phylogenetic analyses

Sequenced DNA fragments from the present study (after discarding primer sequences and ambiguously aligned regions) and other stunt nematode sequences obtained from GenBank were used in the phylogenetic reconstruction. Outgroup taxa for each dataset were selected based on previously published studies (Handoo et al., 2014; Munawar et al., 2021; Nguyen et al., 2019). Multiple sequence alignments of the newly obtained and published sequences were made using the FFT-NS-2 algorithm of MAFFT V.7.450 (Katoh et al., 2019). Sequence alignments were visualized with BioEdit (Hall, 1999) and manually edited by Gblocks ver. 0.91b (Castresana, 2000) in the Castresana Laboratory server (http://molevol.cmima.csic.es/castresana/Gblocks_server.html) using options for a less stringent selection (minimum number of sequences for a conserved or a flanking position: 50% of the number of sequences + 1; maximum number of contiguous non-conserved positions: 8; minimum length of a block: 5; allowed gap positions: with half).

Phylogenetic analyses of the sequence datasets were conducted based on Bayesian inference (BI) using MRBAYES 3.2.7a (Ronquist and Huelsenbeck, 2003). The best-fit model of DNA evolution was calculated with the Akaike information (AIC) of JMODELTEST V.2.1.7 (Darriba et al., 2012). The best-fit model, the base frequency, the proportion of invariable sites, substitution rates and the gamma distribution shape parameters in the AIC were used for phylogenetic analyses. BI analyses were performed under a transitional model, with a rate of variation across sites (TIM3 + G) for the partial 28S region; a transversional model with a proportion of invariable sites and a rate of variation across sites (TVM + I + G) for ITS; and a transitional model with a proportion of invariable sites and a rate of variation across sites (TIM1ef + I + G) for 18S region. These BI analyses were run separately per dataset with four chains for 2 × 10^6^ generations. The Markov chains were sampled at intervals of 100 generations. Two runs were conducted for each analysis. After discarding burn-in samples of 30% and evaluating convergence, the remaining samples were retained for more in-depth analyses. The topologies were used to generate a 50% majority-rule consensus tree. Posterior probabilities (PP) are given on appropriate clades. Trees from all analyses were edited by FigTree software V.1.4.4 (Rambaut, 2014).

## Results and description

### Systematics

**Figure 1:**
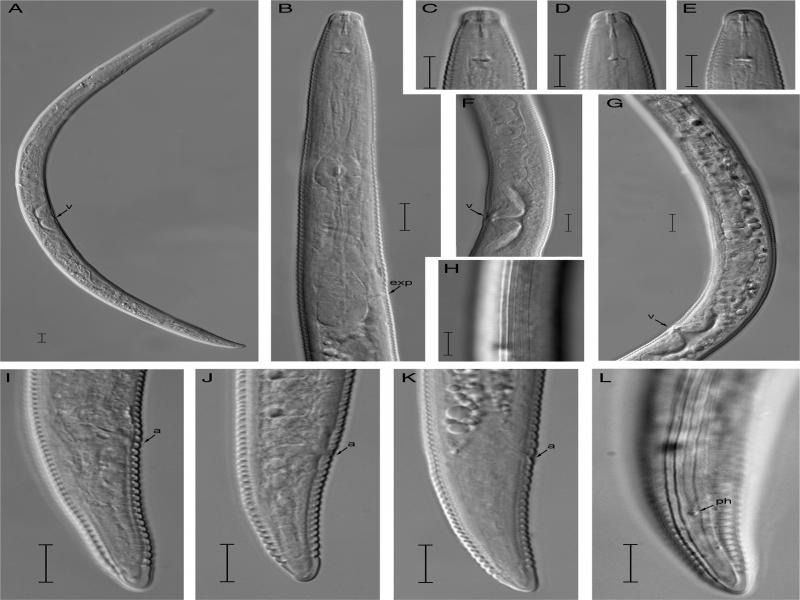
*Quinisulcius curvus* Female. (A) Entire female; (B) Pharyngeal region; (C-E) Lip region; (F, G) Vulval region; (H) Lateral lines; (I-L) Tail regions. Scale bars (A = 10 μm; B-L = 10 μm) Abbreviation: a=anus; exp = excretory pore; ph = phasmid; v = vulva.

**Figure 2:**
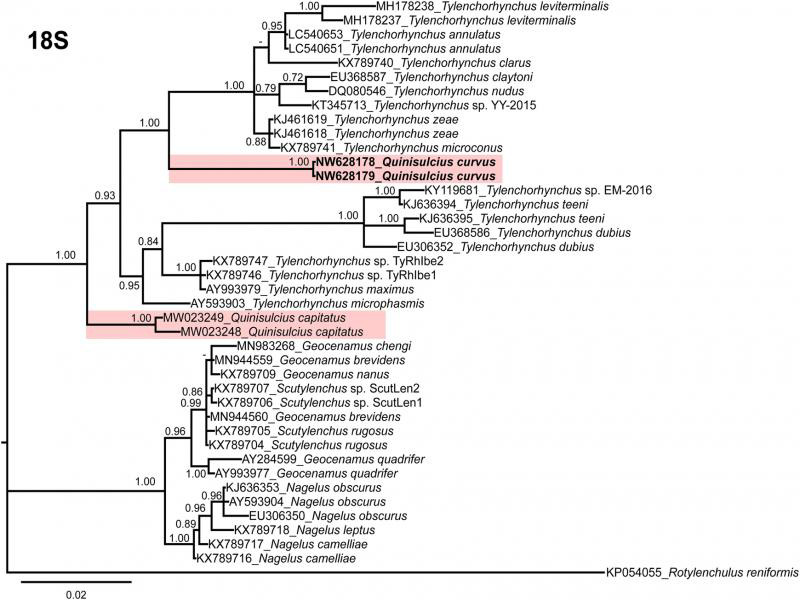
Phylogenetic relationships within selected genera of subfamily Telotylenchinae and subfamily Merliniinae as inferred from Bayesian analysis using the 18S of the rRNA gene sequence dataset with the TIM1ef + I + G model. Posterior probability of more than 70% is given for appropriate clades. Newly obtained sequences are indicated in bold.

**Figure 3:**
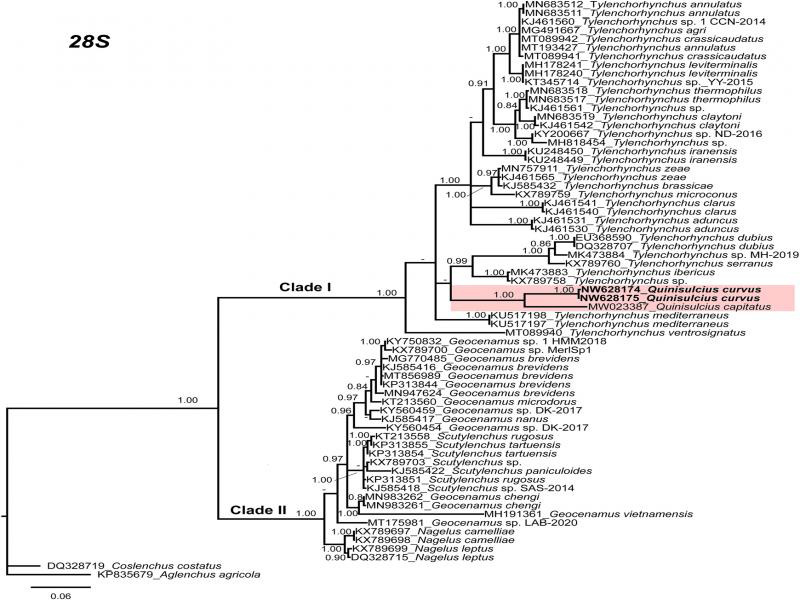
Phylogenetic relationships within selected genera of subfamily Telotylenchinae and subfamily Merliniinae as inferred from Bayesian analysis using the 28S of the rRNA gene sequence dataset with the TIM3 + G model. Posterior probability of more than 70% is given for appropriate clades. Newly obtained sequences are indicated in bold.

**Figure 4:**
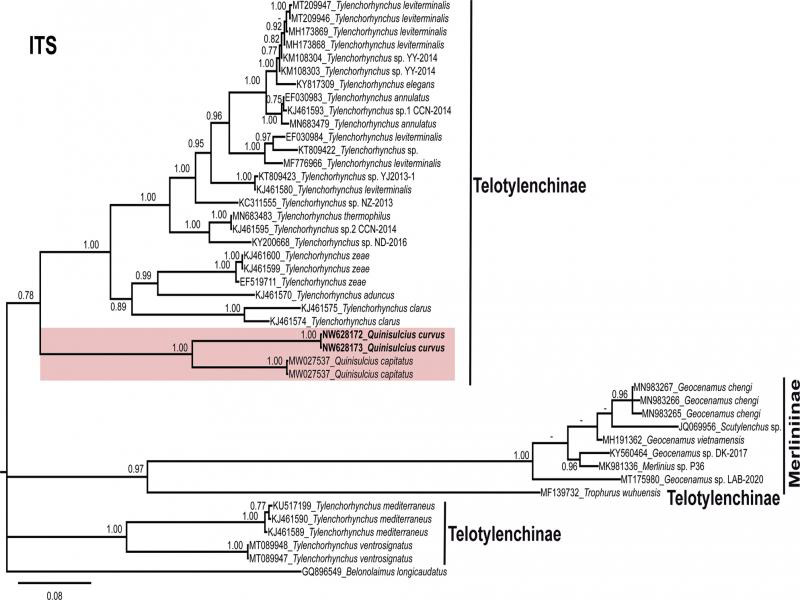
Phylogenetic relationships within selected genera of subfamily Telotylenchinae and subfamily Merliniinae as inferred from Bayesian analysis using the ITS of the rRNA gene sequence dataset with the TVM3 + I + G model. Posterior probability of more than 70% is given for appropriate clades. Newly obtained sequences are indicated in bold.

***Quinisulcius curvus*** (Williams, 1960) Siddiqi, 1971

(*=Tylenchorhynchus curvus* Williams, 1960)

(Figures 1–4; Table 1)

**Table 1.** Comparative morphometrics of *Quinisulcius curvus* (Williams, 1960) Siddiqi, 1971.

	This study	Williams, 1960	Mizukubo et al., 1993	Li et al., 1986
Origin	Hainan, China	Mauritius	Thailand	Henan, China
Host	*Guettarda speciosa*	Sugarcane	Maize	*Brassica caulirapa*
Male	Unknown	Unknown	Unknown	Unknown
n	20	-	6	1
L	581 ± 36.7 (503-644)	490-630	547 ± 56.9 (442-606)	603.2
a	27.2 ± 1.6 (24.3-31.0)	29-37	28.5 ± 2.37 (25.7-31.8)	27.3
b	5.0 ± 0.3 (4.6-5.6)		5.3 ± 0.18 (5.0-5.5)	4.9
c	16.5 ± 0.7 (15.3-18.0)	15-18	17.3 ± 1.19 (15.4-18.9)	16.6
c'	2.5 ± 0.2 (2.3-2.9)	2.5	2.5 ± 0.10 (2.3-2.6)	-
V	56.9 ± 1.6 (53.6-60.0)	52-57	56.4 ± 0.7 (55.3-57.2)	55.2
MB (median bulb position)^a^	54.8 ± 11.7 (50.7-93.5)	51	51.0 ± 2.2 (49.2-55.3)	-
Lip height	3.0 ± 0.3 (2.6-3.5)	-	-	-
Lip width	6.6 ± 0.4 (5.7-7.2)	-	-	-
Lip diam./lip height	2.2 ± 0.2 (1.8-2.5)	-	-	-
Stylet length	12.6 ± 0.5 (11.5-13.5)	17	14.6 ± 0.60 (13.5-15)	18.2
Conus length	5.2 ± 0.3 (4.5-6.0)	-	-	-
DGO	2.1 ± 0.3 (1.7-2.6)	1.5	1.4 ± 0.2 (1.2-1.7)	-
Median bulb length	13.4 ± 0.5 (12.4-14.2)	-	-	-
Median bulb diam.	10.1 ± 0.4 (9.6-11.0)	-	-	-
Median bulb length/diam.	1.3 ± 0.1 (1.2-1.4)	-	-	-
Distance from anterior end to hemizonid	97.7 ± 4.7 (89.0-106.7)	-	-	-
Distance from anterior end to excretory pore	103.6 ± 4.3 (93.2-110.3)	-	99 ± 9.1 (83-106)	-
Maximum body diam.	21.3 ± 0.9 (19.2-23.0)	19	-	-
Vulval body diam.	20.1 ± 0.9 (18.3-22.3)	-	-	-
Anterior reproductive branch	161.1 ± 15.4 (119.7-188.1)	-	-	-
Posterior reproductive branch	157.6 ± 24.7 (109.5-208.0)	-	-	-
Body diam. at anus	14.2 ± 0.7 (13.1-15.7)	-	-	-
Tail length	35.3 ± 2.8 (29.6-40.7)	39	32 ± 1.9 (29-34)	-
Tail annuli	18.0 ± 2.0 (15.0-23.0)	15-20	15-18	16-17
Phasmid to tail terminus	24.2 ± 2.4 (19.3-29.3)	-	-	-

Note: ^a^MB = Distance between anterior end of body and center of median pharyngeal bulb as percentage of pharyngeal length.

**Female**

Body straight to slightly arcuate ventrally after heat fixation. Cuticle annulated and annuli becomes wider (1.0-1.5 µm) at mid-body. Lateral field with 5 incisures, non-areolated, extending about half of maximum body diameter, the middle incisures not extending past the phasmid; lip region hemispherical, bearing 4 to 7 fine annuli, slightly offset from body, 6.0 to 7.0  µm wide, 2.5 to 3.5  µm high; labial framework lightly sclerotized. Stylet knobs rounded, slightly posteriorly directed, 3.0 to 4.0  µm across. Dorsal gland orifice at 1.5 to 2.5  µm behind the stylet knobs. Median bulb oval, with conspicuous valve plates. Isthmus slender encircled with nerve ring. Excretory pore located at the middle of basal pharyngeal bulb, hemizonid 3 to 4 body annuli anterior to excretory pore. Basal pharyngeal bulb pyriform, abutting intestine. Cardia hemispherical. Reproductive system didelphic-amphidelphic, oocytes in single row. Vagina about half vulval body diam. deep, vulva depressed, apparently covered with a flap, spermatheca irregular inconspicuous, without sperm. Anus prominent. Tail cylindrical, with non-annulated bluntly rounded terminus, bearing 15 to 23 annuli on ventral side. Phasmids almost at middle of tail.

### Male

Not found.

### Taxonomic notes

*Quinisulcius curvus* was originally described from Mauritius in the rhizosphere of sugarcane (Williams, 1960). Since then, the species has been reported from Dominican Republic, Pacific Islands, Martinique, Thailand, India, Pakistan in the rhizosphere of sugarcane, grapes, maize, tuber and vegetable crops (Bohra and Baqri, 2005; Bridge, 1988; Cadet et al., 1994; Hussain et al., 2016; Mizukubo et al., 1993; Román, 1968; Toida et al., 1996). Though it has been reported from several countries but morphometrical and morphological data was only provided in few reports (Table 1). Morphometrically, it is observed that the Hainan population of *Q. curvus* has a shorter stylet than the type population (11.5-13.5 µm vs 17.0 µm) and other reported descriptions (Table 1), but close to a Thailand population (11.5-13.5 µm vs 13.5-15 µm) (Mizukubo et al., 1993), suggesting a high intraspecific variability on this character. However, the rest of morphometrics and morphological characters e.g. body habitus, lip and tail morphology, and lateral field characters posterior to the phasmid correspond well with the original description. Vulva and spermatheca morphology was not described in the original or subsequent descriptions. The vulva of the Hainan population has fine lips and apparently covered with a vulval flap. The spermatheca is non-functional, weakly developed and irregularly shaped. No sperm was observed in the spermatheca. Male was not described in the original description or in the subsequent descriptions (Li et al., 1986; Mizukubo et al., 1993; Williams, 1960), same as in the Hainan population. Out of 17 nominal species of *Quinisulcius*, males were not reported for 10 species (Geraert, 2011). It can be speculated that *Q. curvus* is a parthenogenetic species as evidenced by the empty spermatheca.

In broader sense, the species *of Quinisulcius* are medium to large worms; their body length ranges from 460 to 911 µm. The shortest species is *Q. quaidi* (Zarina and Maqbool, 1992) (744-911 µm) and the longest is *Q. capitatus* (830-960 µm). The general body habitus is arcuate to C-shape, however, *Q. seshadrii* (Giribabu and Saha, 2002) has spiral body habitus. The stylets of *Quinisulcius* species are moderately strong, ranging from 11 to 23 µm. The shortest stylet was reported for *Q. quaidi* (11-13 µm) and the longest for *Q. dalatensis* (Nguyen and Nguyen, 1998) (21-23 µm). The excretory pore position is quite variable; however, it is always located in the region of the pharyngeal bulb or anterior to it. The tail is variable, with a length ranging from 30 to 58 µm, the longest of which was reported for *Q. brevistyletus* (Kulinich, 1985) (51-58 µm). The general morphology of the tail is elongate conoid and ventrally curved with rounded to pointed terminus (Geraert, 2011). Our species in question, *Q*. *curvus* is morphologically close to *Q. acutus* and *Q. capitatus*. It can be differentiated from both species by smaller body size, smaller stylet and shape of tail region. *Q. curvus* differs from *Q. acutus* by lip morphology (hemispherical continuous without constriction vs rounded set off by constriction), stylet knobs (rounded vs massive cupped-shaped), cardia shape (hemispherical vs conoid-rounded), shorter body (581 (503-644) µm vs 600-750 µm), and shorter stylet (12.6 (11.5-13.5) µm vs 15-17 µm). From *Q. capitatus,* it can be distinguished by the shape of lip region (hemispherical vs rounded), shorter body (581 (503-644) µm vs 744.0-911.0 µm), and shorter stylet (12.6 (11.5-13.5) µm vs 15.5-20.4 µm).

### Habitat and locality

This population was collected in the rhizosphere of sea randa (*Guettarda speciosa* L.) from Ganquan Island, Sansha City, Hainan Province, China on September 23, 2019 (6.509068N, 111.596901E).

### Molecular characterisation and phylogeny

The sequences of nearly full-length 18S (1649 bp, NW628178-NW628179), ITS region of rDNA (806 bp, NW628172-NW628173) and 28S D2-D3 region (742 bp, NW628174-NW628175) of *Q. curvus* were obtained in the present study. Phylogenetic relationships among the subfamily Merliniinae and subfamily Telotylenchinae nematodes were determined separately for each dataset using BI (Figs. 2-4). These subfamilies were selected because of the genus *Quinisulcius* is morphologically close to both of them.

So far, *Q. capitatus* is the only species of the genus that has been molecularly characterized. The sequence identities of partial 18S, ITS and 28S rDNA of *Q. curvus* with *Q. capitatus* are 95% (34 bp difference, 0 indel), 82% (99 bp difference, 44 indels) and 89% (75 bp difference, 10 indels), respectively, confirming the species separation.

The phylogenetic analyses of *Q. curvus* are presented in Figures 2-4. The trees inferred from 18S, and 28S analyses separated into two distinct clades (PP = 1.00, 1.00, respectively) which comprises species of subfamilies Telotylenchinae and Merliniinae; however, in ITS tree Telotylenchinae appears separate in three different groups (Fig. 4). In all the trees, both *Quinisulcius* species (*Q. capitatus* and *Q. curvus*) are clustered together and grouped with members of Telotylenchinae, except for 18S tree (Fig. 2), in which both species appeared on separate branches among species of *Tylenchorhynchus*. However, in 28S and ITS trees (Figs. 3 and 4), *Q. curvus* and *Q. capitatus* grouped together as a subclade within species of *Tylenchorhynchus*. We also anticipate that with the inclusion of more sequences from *Quinisulcius* and species from other genera within these subfamilies will certainly clarify the phylogenetic positioning of *Q. curvus* and *Q. capitatus*, as well as the monophyletic or paraphyletic status of the genus *Quinisulcius* and the subfamilies Merliniinae and Telotylenchinae.

Our results suggest that Merliniinae and Telotylenchinae appears clearly separated with 18S and 28S ribosomal markers (Figs. 2 and 3), confirming morphological separations, and appearing as monophyletic separated groups. Nevertheless, in ITS Telotylenchinae appears as a polyphyletic group (Fig. 3). Consequently, additional species characterization under integrative taxonomic approaches are needed to confirm this hypothesis by studying several species of other genera within these subfamilies.

### Remarks

The host associations or preference of *Q. curvus* has not been well documented. It was initially reported from the sugarcane rhizosphere in Mauritius (Williams, 1960), subsequently it has been detected in sugarcane growing areas of Martinique and Australia (Nobbs, 2013; Román, 1968). From these reports, it can be speculated that *Q. curvus* has a host preference for sugarcane. In addition to that, *Q. curvus* has also been reported from other countries in the rhizosphere of different horticultural crops, although no considerable plant damage was reported (Table 1). *Quinisulcius curvus* was also reported in Henan Province of China from the rhizosphere of *Brassica caulorapa*, but the diagnosis of this population was based on a single specimen (Li et al., 1986, Table 1). In our opinion, a morphological identification based on single nematode is doubtful. Therefore, the status of the Henan population of *Q. curvus* needs further sampling and confirmation.

It has been noted that *Q. curvus* has always been reported in association with principle agricultural or horticultural crops. In this study, we found *Q. curvus* in the rhizosphere of shrub sea randa i.e. *G. speciosa*, which is a new host record for this species. The discovery of *Q. curvus* from China highlights the need to update the list of distribution of these nematodes. Moreover, comprehensive surveys will likely uncover other *Quinisulcius* species from China.
